# Whole‐body hot water immersion effect on cerebral haemodynamics and subsequent cerebrovascular reactivity to carbon dioxide

**DOI:** 10.1113/EP093072

**Published:** 2025-10-17

**Authors:** Samuel F. Leaney, Ester Tommasini, Guto W. Hughes, Alison M. Shepherd, Nyah D. Kingdon, Justin S. Lawley, Geoff B. Coombs, Jonathan P. Moore, Samuel J. Oliver

**Affiliations:** ^1^ Institute for Applied Human Physiology, School of Psychology and Sport Science, College of Medicine and Health Bangor University Bangor UK; ^2^ Department of Psychology Università Cattolica del Sacro Cuore Milan Italy; ^3^ Department of Sport Science University of Innsbruck Innsbruck Austria

**Keywords:** blood pressure, cerebral blood flow, cerebrovascular function, heat stress, heat therapy

## Abstract

To test the hypothesis that hot water immersion (HWI) improves cerebrovascular function via shear‐mediated mechanisms, this study determined cerebrovascular reactivity to carbon dioxide ($\mathrm{CV}R_{CO_{2}}$) before and after 60 min of 39°C HWI and a 21°C air control (CON) in 15 healthy participants. Thermal and haemodynamic variables were assessed throughout the trials, and $\mathrm{CV}R_{CO_{2}}$ was determined by a 4‐min inhalation of hypercapnic gas (6% CO_2_, 21% O_2_, N_2_ balance) and the assessment of internal carotid artery (ICA) blood flow by duplex ultrasound before and 45 min after HWI and CON. At 60 min of the interventions, core body temperature (CON, 36.9 ± 0.3°C; HWI, 38.1 ± 0.3°C, *P *< 0.01), heart rate (*P *< 0.01) and ICA conductance (*P *< 0.01) were higher in HWI than CON, while, mean arterial blood pressure was lower (CON, 82 ± 9 mmHg; HWI 65 ± 8 mmHg, *P *< 0.01). No differences were observed for ICA diameter, ICA blood velocity, ICA shear rate and ICA blood flow between HWI and CON (all *P *> 0.05). ICA $\mathrm{CV}R_{CO_{2}}$ did not change after either CON (pre: 13.9 ± 9.2 to post: 11.3 ± 6.1 mL min^−1^ mmHg^−1^) or HWI (pre: 14.6 ± 7.9 to post: 10.9 ± 5.4 mL min^−1^ mmHg^−1^; Interaction *P* = 0.65). In conclusion, HWI reduced blood pressure and increased ICA conductance (i.e. autoregulation) to maintain blood flow to the brain; however, HWI did not influence subsequent cerebrovascular function, as assessed by $\mathrm{CV}R_{CO_{2}}$.

## Abstract (highlights)

- **What is the central question of this study?**
  Does a single session of whole‐body hot water immersion increase internal carotid artery blood flow and shear rate, and transiently increase subsequent cerebrovascular function, assessed by volumetric cerebrovascular reactivity to hypercapnia?
- **What is the main finding and its importance?**
  Whole‐body hot water immersion decreased blood pressure and increased internal carotid artery cerebrovascular conductance but not volumetric blood flow or shear rate. Subsequent cerebrovascular reactivity remained unaltered following hot water immersion, indicating no acute influence of heating on volumetrically assessed cerebrovascular function.

## 1 INTRODUCTION

Regular heat therapy has been associated with reduced cardiovascular and cerebrovascular disease risk in epidemiological studies (Laukkanen et al., 2015; Ukai et al., 2020). The lower disease risk is likely multi‐factorial and may partly be explained by enhanced peripheral vascular function and lowered blood pressure (Brunt & Minson, 2021; Pizzey et al., 2021). For example, in a sham‐controlled study, an 8‐week whole body passive hot water immersion (HWI) intervention improved brachial artery endothelium‐dependent function and reduced blood pressure and arterial stiffness (Brunt et al., 2016). These longer‐term adaptations may be explained by shear‐mediated increases in nitric oxide (NO) bioavailability (Brunt et al., 2019; Hoekstra et al., 2018) and reductions in oxidative stress, inflammation (Steward et al., 2024) and circulating endothelial‐derived microvesicles (Bain et al., 2017). Due to physiological differences between the peripheral and cerebrovascular vasculature (Koep et al., 2022), it remains unclear if these mechanisms explain the reduced incidence of cerebrovascular disease observed with regular HWI (Ukai et al., 2020). As brain hypoperfusion precedes cerebrovascular‐related pathology (Fang et al., 2022; Sweeney et al., 2018; Toth et al., 2017), interventions capable of increasing cerebral blood flow (CBF), shear rate and reversing hypoperfusion may be beneficial for cerebrovascular function and health (Calverley et al., 2020; Green et al., 2017; Iwamoto et al., 2020).

Vascular shear‐mediated processes are a potentially important stimulus for improving cerebrovascular function and health. Indeed, interventions that increase cerebrovascular shear rate and CBF have been shown to transiently increase cerebral endothelium‐dependent and ‐independent function immediately after a single exercise session (Sakamoto et al., 2024) and intermittent hypoxia (Iwamoto et al., 2020), respectively. Repetitive exposure to cerebral shear and CBF increasing interventions may contribute to longer‐term improvements in cerebrovascular function (Murrell et al., 2013). As HWI has been shown to increase CBF during moderate hyperthermia (+1°C core temperature) and increase ICA shear rate (ICA_SR_) (Gibbons et al., 2021; Worley et al., 2025), it is plausible that HWI may improve subsequent cerebrovascular function.

Cerebrovascular reactivity to carbon dioxide ($\mathrm{CV}R_{CO_{2}}$), the change in CBF per unit change in end‐tidal carbon dioxide, is a non‐selective endothelium‐independent assessment of cerebral vasoreactivity and may be used as an index of cerebrovascular function (Fierstra et al., 2013; Hoiland et al., 2022; Sleight et al., 2021). $\mathrm{CV}R_{CO_{2}}$ reductions are observed with ageing (Peng et al., 2018) and predict cerebrovascular disease in patients with carotid artery disease (Markus & Cullinane, 2001). Conversely, $\mathrm{CV}R_{CO_{2}}$ is higher in aerobically fit than unfit older adults (Barnes et al., 2013) and can be improved after 12 weeks of regular aerobic exercise (Murrell et al., 2013). Two studies have assessed $\mathrm{CV}R_{CO_{2}}$ after HWI, demonstrating that HWI does not acutely alter $\mathrm{CV}R_{CO_{2}}$ (Worley et al., 2021, 2025). In these studies, $\mathrm{CV}R_{CO_{2}}$ was measured in the middle cerebral artery (MCA) by transcranial Doppler ultrasound (TCD). This method of $\mathrm{CV}R_{CO_{2}}$ does not incorporate the MCA diameter. As MCA diameter increases during even modest steady state hypercapnia (+4 mmHg $P_{\mathrm{ETC}O_{2}}$) (Al‐Khazraji et al., 2021), MCA blood velocity (MCA_V_) alone does not accurately characterise CBF during MCA $\mathrm{CV}R_{CO_{2}}$. This can be achieved by Duplex ultrasound, which concurrently measures blood velocity and blood vessel diameter to enable calculations of volumetric CBF. Moreover, the assessment of volumetric ICA $\mathrm{CV}R_{CO_{2}}$ after HWI is particularly warranted as Worley et al. (2025) observed an increased ICA blood flow (ICA_Q_) but not MCA_V_ during their 39°C HWI protocol.

The purpose of this study was to examine the cerebrovascular haemodynamic responses to a single HWI. Based on previous research showing that HWI increases ICA_Q_ and ICA_SR_ (Gibbons et al., 2021; Worley et al., 2025), and as cerebrovascular function is improved after other cerebral shear rate increasing interventions, such as aerobic exercise (Sakamoto et al., 2024) and intermittent hypoxia (Iwamoto et al., 2020), we hypothesised that HWI would elevate ICA_Q_ and ICA_SR_ for the duration of the HWI and improve subsequent cerebrovascular function, measured by an increase in volumetrically calculated ICA $\mathrm{CV}R_{CO_{2}}$ post‐HWI.

## 2 METHODS

### 2.1 Ethics and informed consent

Ethical approval was obtained from Bangor University, School of Psychology and Sport Science Academic Research Ethics Committee (no. 2024‐0257‐2) for this study, which was conducted following the standards of the *Declaration of Helsinki*, except for registration in a database, with written and informed consent obtained from all study volunteers.

### 2.2 Participants

Fifteen participants were recruited for the study (6 female, 9 male; age 28 ± 4 years, height 174 ± 8 cm, body mass 74 ± 14 kg, body fat 17 ± 8%, physical activity 4474 ± 2542 MET min week^−1^). An a priori sample size was estimated (G*Power version 3.1.9.4: Heinrich Heine University Düsseldorf, Düsseldorf, Germany) as adequate to detect a significant difference in cerebrovascular function between HWI and control trials, using standard α (0.05), power (0.80), and the effect size ƒ = 0.33. This effect size was based on the effect size (ƞ^2^ = 0.34) reported by Iwamoto et al. (2020) for increased cerebrovascular function (hypercapnia‐induced vasodilatation of the ICA) after an ICA shear‐increasing intervention. To account for differences in our intervention and main outcome measure, a more conservative ƞ^2^ = 0.1 was used, and ƒ = 0.33 was calculated using the formula: $f=\sqrt{\eta^{2}/\left(1-\eta^{2}\right)}\;$ (Cohen, 1988). Fifteen participants were recruited to allow for dropout. All participants were healthy, non‐smokers, and free from known cardiovascular, neurological, respiratory or metabolic disease. Participants also had no history of heat illness and were not engaged in any form of whole‐body heat exposure, for example, HWI or sauna, more than once per week. To account for fluctuations in body temperature (Baker et al., 2020) and vascular function (Williams et al., 2020), female participants were studied in the same phase of their menstrual cycle (4 early follicular phase, 1 luteal phase, 1 contraceptive implant).

### 2.3 Study design

**FIGURE 1:**
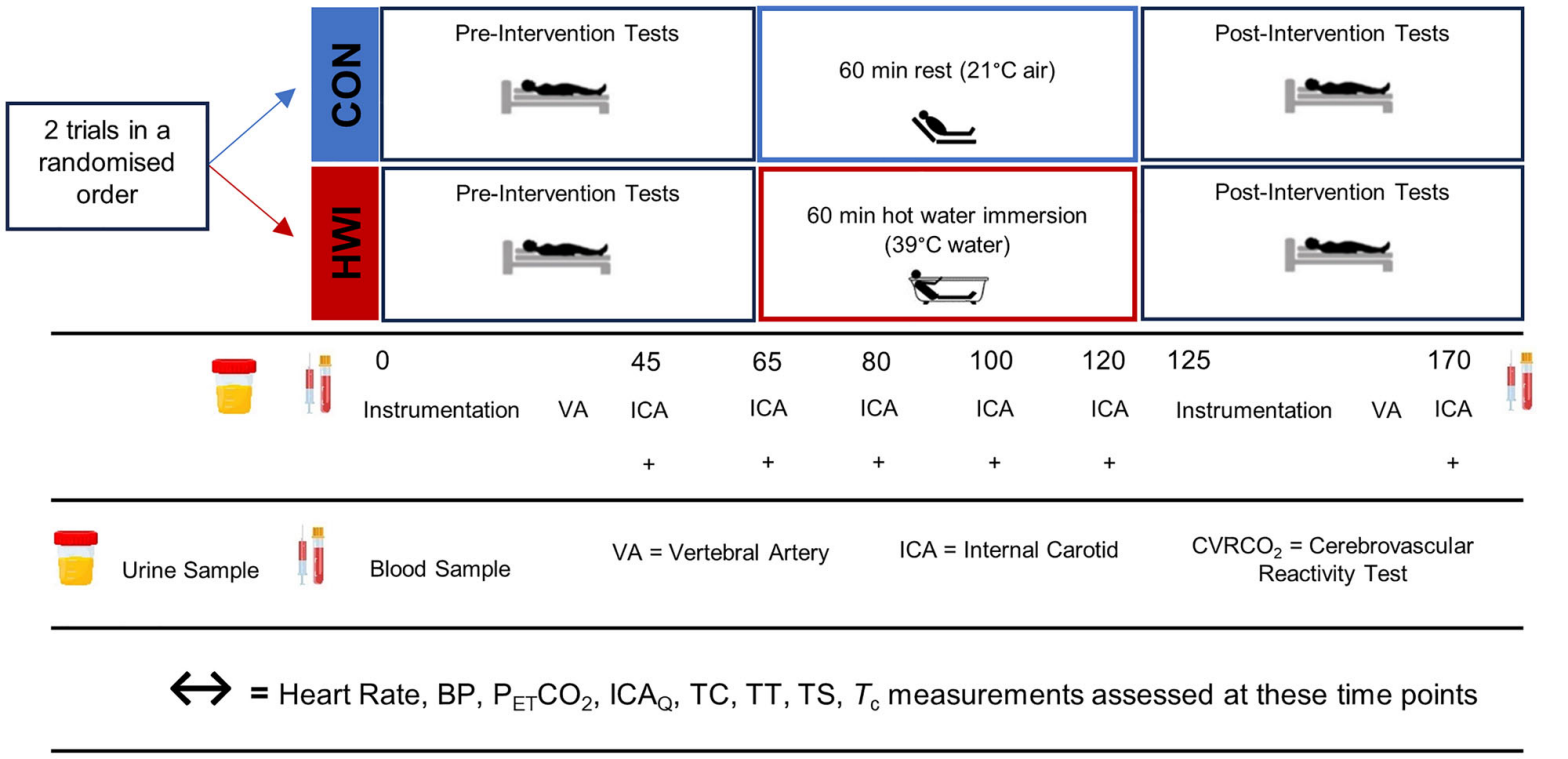
Experimental protocol. BP, blood pressure; CON, control trial; HWI, hot water immersion trial; ICA_Q_, internal carotid artery blood flow; $P_{\mathrm{ETC}O_{2}}$, partial pressure of end‐tidal carbon dioxide; *T*_c_, core temperature; TC, thermal comfort; TS, thermal sensation; TT, thermal tolerance.

Participants visited the laboratory on three occasions for familiarisation and two subsequent experimental trials. A randomised, crossover design was used with each participant completing both experimental trials separated by at least 48 h. Experimental trials consisted of either 60 min of HWI or a time and position‐matched control in ambient room air (CON). Participants completed the two experimental trials in the morning at the same time of day (∼09.00 h) and followed the same experimental procedures (Figure 1).

### 2.4 Familiarisation

Before the two experimental visits, a familiarisation session was conducted to accustom participants to the laboratory environment and experimental trials, and screen participants’ ICA and vertebral arteries (VA). During this visit, participants' height was determined by a wall‐mounted measuring tape, and body mass and composition (fat‐free mass, fat mass) by specialised bio‐electrical impedance analysis weighing scales (Tanita, Manchester, UK). Physical activity was assessed using a self‐reported activity form (Craig et al., 2003).

### 2.5 Experimental interventions (HWI and CON)

During the HWI trial, participants wearing swimsuits entered a pre‐filled hot bath (LUMI recovery PRO, Exeter, UK) that was set and maintained at 39.0 ± 0.2°C. Participants positioned themselves in a semi‐recumbent position to ensure the water level reached their neck with arms under the water. Participants repositioned themselves with shoulders and arms out of the water for blood pressure measurements (5, 20, 40 and 60 min) only. Participants remained in the hot bath for 60 min. During the CON trial, dressed in shorts and a T‐shirt, participants rested in a semi‐recumbent position in a temperate laboratory (temperature 21.1 ± 1.4°C, relative humidity 55 ± 7%, pressure 759 ± 6 mmHg). Participants were allowed to drink water ad libitum and were provided water to consume after HWI to replace fluid losses. During both interventions, heart rate, blood pressure, ICA haemodynamics and perceptual thermal measures, by standardised scales (International Organization for Standardization, 2019), were quantified after 5 min and at 20‐min intervals (time points: 5, 20, 40 and 60 min) throughout both experimental interventions (Figure 1).

### 2.6 Experimental procedures

Participants arrived having refrained from heat exposure for ≥36 h, strenuous exercise for ≥24 h, alcohol and caffeine for ≥12 h and food for ≥6 h. This standardisation is aligned with technical recommendations for extracranial artery blood flow assessment (Thomas et al., 2015). Before experimental trials began, participants provided a small urine sample (<25 mL) to confirm their hydration status with a urine osmolality reader (Pocket PAL‐OSMO, Vitech Scientific, West Sussex, UK).

To begin, participants rested supine in an air‐conditioned laboratory (temperature 21.7 ± 0.7°C, relative humidity 56 ± 6%, pressure 759 ± 5 mmHg) and were instrumented for continuous physiological monitoring. Heart rate and blood pressure were recorded with a lead II electrocardiogram and finger photoplethysmography, respectively (Finometer Midi, Finapres Medical Systems, Enschede, Netherlands). Measurements of systolic blood pressure (SBP), diastolic blood pressure (DBP) and mean arterial pressure (MAP) were calculated from the Finometer output (reconstructed brachial artery pressure waveform) and calibrated to the median of three automated brachial blood pressure measurements (Tango+, SunTech, Morrisville, NC, USA). The partial pressure of end‐tidal oxygen ($P_{\mathrm{ET}O_{2}}$) and carbon dioxide ($P_{\mathrm{ETC}O_{2}}$) were recorded from a mask fitted to the participant connected to a breath‐by‐breath gas analyser (ML206, ADInstruments, Colorado Springs, CO, USA). Core temperature (*T*_c_) was measured using a rectal thermistor self‐inserted 15 cm beyond the anal sphincter (YSI 4000A, Dayton, OH, USA) and skin temperature (*T*_sk_) using skin thermistors placed on four skin sites (thigh, calf, chest, upper arm) (VMS‐LDF, Moor Instruments, Axminster, UK). Mean *T*_sk_ was calculated using the following four‐site equation (Ramanathan, 1964):

$$T_{\mathrm{sk}}=0.3\left(T_{\mathrm{chest}}+T_{\mathrm{arm}}\right)+0.2\left(T_{\mathrm{thigh}}+T_{\mathrm{calf}}\right)$$

Mean body temperature (MBT) was calculated using the following formula (Burton, 1935; Lenhardt & Sessler, 2006):

$$\mathrm{MBT}=\left(0.64\times T_{c}\right)+\left(0.36\times T_{\mathrm{sk}}\right)$$

Finally, a 2 MHz TCD probe was placed over the transtemporal window secured in place via an adjustable headpiece (PMD150, Spencer Technologies, Seattle, WA, USA) for the assessment of MCA_V_.

Once instrumented and having rested supine for ∼15 min, brachial artery blood pressure measures were recorded. VA diameter and blood velocity measurements were obtained (∼35 min), followed by ICA diameter and velocity and $\mathrm{CV}R_{CO_{2}}$ (∼45 min) using duplex ultrasound. MCA_V_ was recorded continuously using TCD. $\mathrm{CV}R_{CO_{2}}$ was performed before and 45 min after the experimental intervention (HWI or CON). Hypercapnia for the $\mathrm{CV}R_{CO_{2}}$ was induced using a fixed concentration of 6% inspired CO_2_, 21% O_2_ with nitrogen balance, for 4 min to measure the haemodynamic responses of the ICA and MCA. A facemask connected to a three‐way non‐rebreathing Hans Rudolph valve allowed participants to be switched from room air to the 6% hypercapnic gas mixture using a 125 L Douglas bag.

A venous blood sample was obtained before and after HWI and CON for the determination of haematocrit (Hct) using a microhaematocrit reader (Hawksley, Brighton, UK) and haemoglobin (Hb) using a haemoglobinometer (EKF‐diagnostics Hemo Control, Barleben, Germany). Nude body mass was obtained before and after both interventions to quantify sweat loss corrected for fluid intake. Relative change in plasma volume (%∆PV) was calculated using the following equation (Dill & Costill, 1974; Harrison et al., 1982):

$$\begin{array}{ccc} \%\Delta \mathrm{PV} & = & \left[\left(Hb_{\mathrm{pre}}/Hb_{\mathrm{post}}\right)\times \left(\left(100-\mathrm{Hc}t_{\mathrm{post}}\right)/\left(100-\mathrm{Hc}t_{\mathrm{pre}}\right)\right)-1\right]\\ & & \times \;100 \end{array}$$

### 2.7 Cerebral ultrasound acquisition and analysis

Duplex ultrasound was conducted with a 15 MHz linear transducer (uSmart 3300, Terason, Burlington, MA, USA) to quantify volumetric blood flow of the extracranial cerebral arteries (ICA_Q_ and VA_Q_). High‐resolution images of vessel diameter were acquired using B‐mode imaging, whilst pulse wave mode was used to simultaneously measure the Doppler velocity spectra. The position of measurement in the vessel was chosen on an individual basis to ensure the most reproducible scans could be acquired within subjects and between visits. Care was taken to ensure the strongest Doppler velocity spectrum signal by positioning the Doppler gate in the centre of the artery with a 60° angle of insonation and adjusting to fill the artery lumen following technical guidelines (Thomas et al., 2015). The ICA was measured at least 1.0–1.5 cm distal to the carotid bifurcation, and the VA was measured between C3 and the subclavian artery. Extracranial arteries were scanned on either the left or right sides of the neck (determined based on the clearest image quality during familiarisation) and kept constant for subsequent trials. The scanning order for CBF measures was VA and then ICA in all cases. Insonation of each artery was achieved with probe position, signal depth and gain settings recorded to replicate the placement within and between visits. Continuous beat‐to‐beat measurements of MCA_V_ were acquired continuously at 1 kHz.

Mean vessel diameter and the time‐averaged mean blood velocity (TAMV) of the Doppler spectrum Duplex ultrasound data were captured continuously using custom software (Amin et al., 2021) and sent to an analog‐to‐digital converter (Powerlab 16/35; ADInstruments) interfaced with LabChart (LabChart 8, ADInstruments). Values were taken from at least 10 cardiac cycles to minimise the influence of respiration.

Blood flow ($\dot{Q}$) was calculated using the following formula:

$$\begin{array}{ccc} \dot{Q}\left(\mathrm{ml}\;\mathrm{mi}n^{-1}\right) & = & \left[\pi \times {\left(\mathrm{mean}\mathrm{artery}\mathrm{diameter}\left(\mathrm{cm}\right)/2\right)}^{2}]\times [\mathrm{TAMV}\left(\mathrm{cm}\;s^{-1}\right)\right]\\ & & \times \;60 \end{array}$$

Cerebrovascular conductance (CVC) was calculated for the ICA and MCA with the following formulas:

$$\mathrm{IC}A_{\mathrm{CVC}}\left(\mathrm{ml}\;\mathrm{mi}n^{-1}\mathrm{mmH}g^{-1}\right)=\mathrm{IC}A_{Q}\left(\mathrm{ml}\;\mathrm{mi}n^{-1}\right)/\mathrm{MAP}\left(\mathrm{mmHg}\right)$$

$$\mathrm{MC}A_{\mathrm{CVC}}\left(\mathrm{cm}\;s^{-1}\mathrm{mi}n^{-1}\;\mathrm{mmH}g^{-1}\right)=\mathrm{MC}A_{V}\left(\mathrm{cm}\;s^{-1}\right)/\mathrm{MAP}\left(\mathrm{mmHg}\right)$$

Assuming blood as a Newtonian fluid, with constant viscosity, steady laminar flow and a straight inelastic vessel, Poiseuille's law was applied to determine shear rate as follows (Papaioannou & Stefanadis, 2005):

$$\mathrm{Shear}\mathrm{Rate}\left(s^{-1}\right)=\left[8\times \mathrm{TAMV}\left(\mathrm{cm}\;s^{-1}\right)/\mathrm{mean}\;\mathrm{artery}\;\mathrm{diameter}\left(\mathrm{cm}\right)\right]$$

Global cerebral blood flow (gCBF) was calculated as the sum of two times ICA_Q_ and VA_Q_ (Gibbons et al., 2021):

$$\mathrm{gCBF}\left(\mathrm{ml}\;\mathrm{mi}n^{-1}\right)=2\times \left(\mathrm{IC}A_{Q}+VA_{Q}\right)$$

The $\mathrm{CV}R_{CO_{2}}$ was calculated as the absolute change in ICA_Q_ per unit change (mmHg) in $P_{\mathrm{ETC}O_{2}}$. The absolute $\mathrm{CV}R_{CO_{2}}$ response was calculated for the ICA and MCA using the following equations:

$$\mathrm{ICA}\;\mathrm{CV}RCO_{2}=\frac{\mathrm{Maximum}\;\mathrm{IC}A_{Q}-\mathrm{Baseline}\;\mathrm{IC}A_{Q}}{\mathrm{Maximum}\;\;P_{\mathrm{ETC}O_{2}}-\mathrm{Baseline}\;P_{\mathrm{ETC}O_{2}}}$$

$$\mathrm{MCA}\;\mathrm{CV}RCO_{2}=\frac{\mathrm{Maximum}\;\mathrm{MC}A_{V}-\mathrm{Baseline}\;\mathrm{MC}A_{V}}{\mathrm{Maximum}\;P_{\mathrm{ETC}O_{2}}-\;\mathrm{Baseline}\;P_{\mathrm{ETC}O_{2}}}$$

To account for any influence of blood pressure during $\mathrm{CV}R_{CO_{2}}$, cerebrovascular conductance reactivity was also calculated for the ICA and MCA with the following equations:

$$\mathrm{IC}A_{\mathrm{CVC}}\;\mathrm{CV}RCO_{2}=\frac{\mathrm{Maximum}\;\mathrm{IC}A_{\mathrm{CVC}}-\mathrm{Baseline}\;\mathrm{IC}A_{\mathrm{CVC}}}{\mathrm{Maximum}\;P_{\mathrm{ETC}O_{2}}-\mathrm{Baseline}\;P_{\mathrm{ETC}O_{2}}\;}$$

$$\mathrm{MC}A_{\mathrm{CVC}}\;\mathrm{CV}RCO_{2}=\frac{\mathrm{Maximum}\;\mathrm{MC}A_{\mathrm{CVC}}-\mathrm{Baseline}\;\mathrm{MC}A_{\mathrm{CVC}}}{\mathrm{Maximum}\;P_{\mathrm{ETC}O_{2}}-\;\mathrm{Baseline}\;P_{\mathrm{ETC}O_{2}}}$$

The $\mathrm{CV}R_{CO_{2}}$ baseline values were calculated during the 30 s of supine rest immediately preceding hypercapnia, whilst the maximum values were taken from the highest 30‐s average for ICA_Q_ and MCA_V_ wherever it occurred during the 4‐min hypercapnic period. This approach was chosen as recent data suggest better within and between‐day reliability of $\mathrm{CV}R_{CO_{2}}$ when maximum values are obtained as a dynamic peak, rather than using set time points (Koep et al., 2022).

### 2.8 Statistical analysis

Statistical analysis was conducted using GraphPad Prism version 9 (GraphPad Software, Boston, MA, USA), values are means ± SD unless otherwise stated, and statistical significance was set at *P* < 0.05. To determine any differences in cerebrovascular, cardiorespiratory, perceptual and thermoregulatory data during HWI and CON interventions, a linear mixed effects model (LMM) with Greenhouse–Geisser correction and Tukey‐corrected multiple comparisons was used. Fixed effects of interest were Time (5, 20, 40, 60 min), Trial (CON and HWI) and interaction (Time × Trial), with subject added as a random effect. Student's paired samples *t*‐test was conducted to compare CON and HWI trials on pre‐intervention baseline cardiovascular, haematological and urine values, and sweat loss, change in nude body mass, change in plasma volume, and change in Hb and Hct following both CON and HWI interventions. Cardiorespiratory and cerebrovascular variables during cerebrovascular reactivity assessment were analysed by LMM with Greenhouse–Geisser correction. Fixed effects of interest were Trial (CON × HWI), Time (Pre × Post), Hypercapnia (Baseline × CO_2_) and interaction (Trial × Time × Hypercapnia). $\mathrm{CV}R_{CO_{2}}$ values were analysed by LMM with Greenhouse–Geisser correction and Tukey‐corrected multiple comparisons. Fixed effects of interest were Time (Pre and Post), Trial (CON and HWI) and interaction (Time × Trial).

## 3 RESULTS

Baseline cardiovascular, haematological and urine responses were comparable between the two trials (all *P* > 0.05) and therefore combined as part of the baseline participant characteristics (Table 1). One male participant was excluded from all MCA_V_ data analysis due to poor signal quality. One female participant was excluded from pre‐intervention $\mathrm{CV}R_{CO_{2}}$ analysis due to poor ICA signal quality, but their remaining three $\mathrm{CV}R_{CO_{2}}$ assessments were kept in the final data set. Blood pressure values from one male participant were removed from the pre‐intervention CON trial $\mathrm{CV}R_{CO_{2}}$ assessment due to a finger photoplethysmography equipment malfunction. ICA_Q_ data was excluded from one male participant at the 20‐min time point during HWI due to poor signal quality.

**TABLE 1:** Anthropometric, body composition, resting physiology and physical activity characteristics.

Variable	Value
Sex (male:female)	9:6
Age (years)	28 ± 4
Height (cm)	174 ± 8
Body Mass (kg)	74 ± 14
Body surface area (m^2^)	1.9 ± 0.2
BMI (kg m^2^)	24 ± 3
Body Fat (%)	17.2 ± 8.1
Hb (g dL^−1^)	14.9 ± 1.5
Hct (%)	45.0 ± 3.5
Urine osmolality (mOsm kg^−1^)	533 ± 276
SBP (mmHg)	119 ± 11
DBP (mmHg)	68 ± 7
MAP (mmHg)	85 ± 7
Heart Rate (bpm)	61 ± 10
PA (MET min week^−1^)	4474 ± 2542

*Note*: Data are presented as means ± SD. *n* = 15. Abbreviations: BMI, body mass index; DBP, diastolic blood pressure; Hb, haemoglobin; Hct, Haematocrit; MAP, mean arterial blood pressure; PA, physical activity level; SBP, systolic blood pressure.

### 3.1 Thermal, cardiorespiratory and cerebrovascular responses to 60 min in 21°C air (CON) and 39°C HWI

**FIGURE 2:**
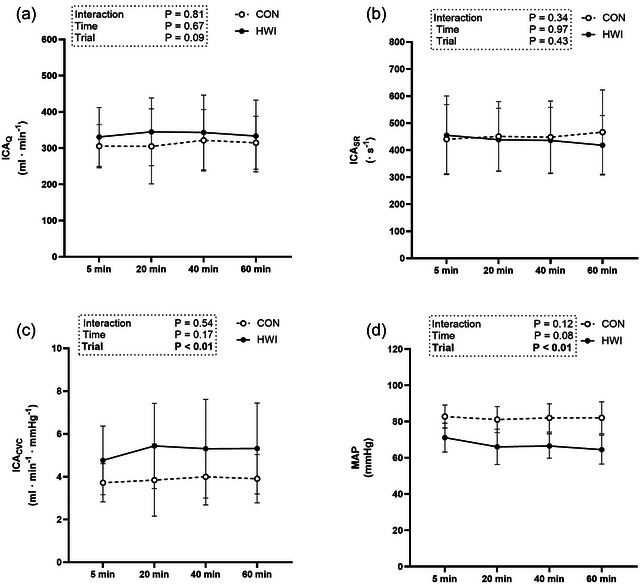
Internal carotid artery blood flow (ICA_Q_, a), internal carotid artery shear rate (ICA_SR_, b), internal carotid artery cerebrovascular conductance (ICA_CVC_, c) and mean arterial blood pressure (MAP, d) at 5, 20, 40 and 60 min time points in 21°C air control (CON) and 39°C hot water immersion (HWI). Data are presented as means (circles) ± standard deviation (bars) for CON (open circles, dotted line) and HWI (filled circles, continuous line). Analysed by linear mixed effects model (LMM) with Greenhouse–Geisser correction to assess the effect of Time (Pre and Post), Trial (CON and HWI) and interaction (Time × Trial). All data are *n* = 15.

During HWI, *T*_c_, heart rate, and thermal sensation were higher, and thermal comfort and tolerance were lower than CON (trial effect *P* < 0.01, Table 2). Blood pressures (SBP, DBP and MAP) were lower during HWI than CON (trial effect *P* < 0.01, Table 2), with the effect on DBP more pronounced at 40 and 60 min into HWI. During HWI $P_{\mathrm{ETC}O_{2}}$ was lower than CON at 40 and 60 min (interaction effect *P* < 0.01, Table 2). In addition, $P_{\mathrm{ETC}O_{2}}$ was lower at 20 min than 40 min in CON only. No main effects of trial were observed for $P_{\mathrm{ETC}O_{2}}$. No differences were detected between HWI and CON for ICA_Q_ (Figure 2a), ICA blood velocity, ICA_SR_ (Figure 2b) or ICA diameter (Table 2) (all *P* > 0.05). During HWI, ICA_CVC_ was higher compared to CON (trial effect *P* < 0.01, Figure 2c).

**TABLE 2:** Thermal, cardiorespiratory, and cerebrovascular responses to 60 min in 21°C air (CON) and 39°C HWI.

Variable	Trial	5 min	20 min	40 min	60 min	*P*		
						Time × Trial	Time	Trial
*T*_c_ (°C)	CON	36.8 ± 0.3	36.9 ± 0.3	36.9 ± 0.3	36.9 ± 0.3	**<0.01**	**<0.01**	**<0.01**
	HWI	36.9 ± 0.2	37.3 ± 0.3^*#^	37.7 ± 0.3^*#^	38.1 ± 0.3^*#^			
Thermal comfort	CON	0.0 ± 0.1	0.0 ± 0.1	0.0 ± 0.1	0.0 ± 0.1	**<0.01**	**<0.01**	**<0.01**
	HWI	0.1 ± 0.3	0.5 ± 0.4^*#^	0.9 ± 0.7^*#^	1.1 ± 0.9^*#^			
Thermal sensation	CON	−0.3 ± 0.6	−0.2 ± 0.4	0.0 ± 0.1	0.0 ± 0.1	**<0.01**	**<0.01**	**<0.01**
	HWI	1.3 ± 0.7^#^	1.8 ± 0.6^*#^	2.2 ± 0.7^*#^	2.1 ± 0.7^*#^			
Thermal tolerance	CON	0.0 ± 0.0	0.0 ± 0.1	0.0 ± 0.0	0.0 ± 0.0	**<0.01**	**<0.01**	**<0.01**
	HWI	0.0 ± 0.0	0.4 ± 0.4^*#^	0.7 ± 0.7^*#^	0.9 ± 0.9^*#^			
Heart rate (bpm)	CON	57 ± 9	62 ± 15	59 ± 8	60 ± 10	**<0.01**	**<0.01**	**<0.01**
	HWI	68 ± 12^#^	85 ± 16^*#^	92 ± 17^*#^	96 ± 13^*#^			
MAP (mmHg)	CON	83 ± 6	81 ± 7	82 ± 8	82 ± 9	0.12	0.08	**<0.01**
	HWI	71 ± 8	66 ± 10	67 ± 7	65 ± 8			
SBP (mmHg)	CON	112 ± 9	112 ± 9	112 ± 10	111 ± 11	0.60	0.35	**<0.01**
	HWI	104 ± 11	101 ± 9	101 ± 11	101 ± 9			
DBP (mmHg)	CON	68 ± 7	66 ± 7	67 ± 8	68 ± 9	**0.04**	**0.04**	**<0.01**
	HWI	55 ± 8^#^	51 ± 8^#^	49 ± 6^*#^	48 ± 5^*#^			
$P_{\mathrm{ETC}O_{2}}$ (mmHg)	CON	39.4 ± 3.9	38.2 ± 3.5**^a^**	39.6 ± 3.4	39.2 ± 3.8	**<0.01**	0.27	0.12
	HWI	38.5 ± 3.9	38.7 ± 4.2	37.5 ± 4.3^#^	37.0 ± 4.2^#^			
ICA_v_ (cm s^−1^)	CON	26.7 ± 5.7	27.0 ± 6.2	27.4 ± 6.1	28.1 ± 7.2	0.44	0.99	0.93
	HWI	27.9 ± 7.1	27.7 ± 6.0	27.4 ± 5.7	26.5 ± 5.6			
ICA_D_ (mm)	CON	4.95 ± 0.44	4.88 ± 0.54	5.00 ± 0.55	4.91 ± 0.46	0.41	0.33	0.05
	HWI	5.03 ± 0.56	5.14 ± 0.51	5.15 ± 0.64	5.16 ± 0.55			
ICA_Q_ (ml min^−1^)	CON	306 ± 60	305 ± 103	322 ± 85	315 ± 73	0.81	0.67	0.09
	HWI	331 ± 82	345 ± 93	343 ± 103	333 ± 99			
ICA_SR_ (s^−1^)	CON	440 ± 128	451 ± 129	448 ± 133	467 ± 156	0.34	0.97	0.43
	HWI	455 ± 145	439 ± 116	436 ± 123	418 ± 110			
ICA_CVC_ (ml min^−1^ mmHg^−1^)	CON	3.72 ± 0.89	3.84 ± 1.68	4.00 ± 1.32	3.91 ± 1.13	0.54	0.17	**<0.01**
	HWI	4.77 ± 1.60	5.44 ± 1.99	5.31 ± 2.31	5.32 ± 2.12			

*Note*: Data are presented as means ± SD. All data *n* = 15. Data were collected during 21°C air control (CON) and 39°C HWI trials, at 5, 20, 40 and 60 min time points. Thermal comfort = 0, comfortable to 4, extremely uncomfortable: thermal sensation = −5, extremely cold to 5, extremely hot; thermal tolerance = 0, tolerable to 4, intolerable. Analysed by linear mixed effects model (LMM) with Greenhouse–Geisser correction to assess the effect of Time (5, 20, 40, 60 min), Trial (CON and HWI) and interaction (Time × Trial). *Post hoc* tests using Tukey‐corrected multiple comparisons were used, where ^*^ *P *< 0.05 versus 5min within trial, *^#^P *< 0.05 versus respective CON time point, ^a^ *P* < 0.05 versus 40 min within trial for $P_{\mathrm{ETC}O_{2}}$ only. Abbreviations: DBP, diastolic blood pressure; ICA_CVC_, internal carotid artery cerebrovascular conductance; ICA_D_, internal carotid artery diameter; ICA_Q_, internal carotid artery blood flow; ICA_SR_, internal carotid artery shear rate; ICA_V_, internal carotid artery blood velocity; MAP, mean arterial pressure; $P_{\mathrm{ETC}O_{2}}$, partial pressure of end‐tidal carbon dioxide; SBP, systolic blood pressure; *T*_c_, core temperature.

Sweat loss across the 60‐min intervention was higher in HWI than CON (CON, 0.10 ± 0.14 L; HWI, 0.68 ± 0.40 L, *P* < 0.01). However, due to higher water intake in HWI (0.56 ± 0.10 L) than CON (0 L), the change in nude body mass was not different between trials (CON, −0.07 ± 0.20 kg; HWI, −0.04 ± 0.30 kg, *P = *0.76). The change in Hb was greater in CON than HWI (CON, −0.61 ± 0.58 g dL^−1^; HWI, −1.2 ± 0.65 g dL^−1^, *P = *0.04); however, the change in Hct (CON, −0.66 ± 1.49%; HWI, −0.58 ± 1.05%, *P = *0.86) and change in plasma volume was not different between CON and HWI interventions (CON, 5.8 ± 5.2%; HWI, 2.0 ± 6.1%, *P = *0.08).

### 3.2 Cerebrovascular, cardiorespiratory and thermal responses before and after 60 min in 21°C air (CON) and 39°C HWI

After HWI *T*_c_, *T*_sk_ and MBT were higher than after CON (Table 3, interaction effects, all *P *< 0.05). Apart from heart rate and VA diameter, no other interactions were observed for cerebral and cardiorespiratory responses (Table 3). Specifically, gCBF, ICA_Q_ and VA_Q_ were comparable after CON and HWI interventions (Table 3).

**TABLE 3:** Cerebrovascular, cardiorespiratory and thermal responses pre and post 60 min in 21°C air (CON) and 39°C HWI.

Variable	Trial	Pre	Post	*P*		
				Time × Trial	Time	Trial
gCBF (ml min^−1^)	CON	878 ± 216	893 ± 235	0.84	0.69	0.22
	HWI	838 ± 167	843 ± 221			
gCBF_CVC_ (ml min^−1^ mmHg^−1^)	CON	11.3 ± 3	11.1 ± 3	0.42	0.80	0.33
	HWI	10.5 ± 2	10.9 ± 4			
VA_V_ (cm s^−1^)	CON	13 ± 3	14 ± 3	0.18	0.12	0.18
	HWI	13 ± 2	13 ± 3			
VA_D_ (mm)	CON	3.86 ± 0.39	3.74 ± 0.41	**<0.01**	0.28	0.43
	HWI	3.81 ± 0.41	3.84 ± 0.43			
VA_Q_ (ml min^−1^)	CON	92 ± 31	96 ± 29	0.69	0.50	0.23
	HWI	90 ± 25	91 ± 29			
ICA_V_ (cm s^−1^)	CON	29 ± 7	29 ± 6	0.78	0.60	0.08
	HWI	28 ± 6	27 ± 6			
ICA_D_ (mm)	CON	5.03 ± 0.50	5.08 ± 0.48	0.63	0.14	0.75
	HWI	5.03 ± 0.47	5.12 ± 0.57			
ICA_Q_ (ml min^−1^)	CON	347 ± 97	350 ± 98	0.92	0.84	0.28
	HWI	329 ± 78	331 ± 108			
MCA_V_ (cm s^−1^)	CON	63 ± 18	62 ± 19	0.07	0.06	**0.01**
	HWI	56 ± 16	49 ± 17			
$P_{\mathrm{ETC}O_{2}}$ (mmHg)	CON	39 ± 3	38 ± 3	0.64	**<0.01**	0.14
	HWI	39 ± 4	38 ± 3			
$P_{\mathrm{ET}O_{2}}$ (mmHg)	CON	104 ± 3	106 ± 4	0.38	**<0.01**	0.07
	HWI	105 ± 4	108 ± 5			
Heart rate (bpm)	CON	61 ± 10	58 ± 10^*^	**0.02**	0.59	0.11
	HWI	63 ± 11	65 ± 14^#^			
MAP (mmHg)	CON	79 ± 11	81 ± 9	0.23	0.93	0.95
	HWI	81 ± 9	79 ± 9			
*T*_c_ (°C)	CON	36.7 ± 0.4	36.9 ± 0.3	**<0.01**	**<0.01**	**<0.01**
	HWI	37.0 ± 0.3	37.6 ± 0.2^*#^			
*T*_sk_ (°C)	CON	29.8 ± 1.1	29.8 ± 0.8	**0.03**	0.14	**0.03**
	HWI	29.9 ± 1.2	30.6 ± 1.1^#^			
MBT (°C)	CON	34.3 ± 0.6	34.3 ± 0.4	**<0.01**	**<0.01**	**<0.01**
	HWI	34.4 ± 0.4	35.0 ± 0.3^*#^			

*Note*: Data are presented as means ± standard deviation. *n* = 15 for all variables except MCAv (*n* = 14).

Analysed by linear mixed effects model (LMM) with Greenhouse‐Geisser correction to assess the effect of Time (Pre and Post), Trial (CON and HWI) and interaction (Time × Trial). *Post hoc* tests were Tukey‐corrected multiple comparisons, where: ^*^ *P *< 0.05 versus Pre within trial, ^#^ *P *< 0.05 versus respective CON time point.

Abbreviations: gCBF, global cerebral blood flow; gCBF_CVC_, global cerebrovascular conductance; ICA_D_, internal carotid artery diameter; ICA_Q_, internal carotid artery blood flow; ICA_V_, internal carotid artery blood velocity; MAP, mean arterial pressure; MBT, mean body temperature. MCA_V_, middle cerebral artery blood velocity; $P_{\mathrm{ETC}O_{2}}$, partial pressure of end‐tidal carbon dioxide; $P_{\mathrm{ET}O_{2}}$, partial pressure of end‐tidal oxygen; *T*_c_, core temperature; *T*_sk_, mean skin temperature; VA_D_, vertebral artery diameter; VA_Q_, vertebral artery blood flow; VA_V_, vertebral artery blood velocity.

### 3.3 Cerebrovascular reactivity before and after 60 min in 21°C air (CON) and 39°C HWI

Hypercapnic gas inhalation increased $P_{\mathrm{ETC}O_{2}}$ from ∼40 to ∼52 mmHg, with the change in $P_{\mathrm{ETC}O_{2}}$ not different in CON and HWI (CON, pre: 11.9 ± 2.9 to post: 12.3 ± 3.5 ∆mmHg, *P *= 0.56; HWI, pre: 11.9 ± 2.6 to post: 12.4 ± 3.3 ∆mmHg, *P *= 0.45). Except for $P_{\mathrm{ET}O_{2}}$, the effects of hypercapnia on $P_{\mathrm{ETC}O_{2}}$, heart rate, MAP and cerebrovascular responses did not differ between CON and HWI (Table 4, Trial × Time × CO_2_, all *P* > 0.05).

**TABLE 4:** Cerebrovascular and cardiorespiratory responses to hypercapnia after 60 min in 21°C air (CON) and 39°C HWI.

		Pre		Post	
Variable	Trial	Baseline	Hypercapnia (CO_2_)	Baseline	Hypercapnia (CO_2_)
ICA_Q_ (ml min^−1^)	CON	341 ± 94	491 ± 149	344 ± 100	469 ± 119
	HWI	322 ± 69	489 ± 127	326 ± 109	454 ± 145
	Trial × Time × CO_2_, *P *= 0.21: Trial × Time, *P *= 0.59: Trial × CO_2_, *P *= 0.93: Time × CO_2_, *P *= 0.34: Trial, *P *= 0.25: Time, *P *= 0.66: **CO_2_, *P *< 0.01**				
ICA_V_ (cm s^−1^)	CON	29 ± 8	39 ± 10	28 ± 7	37 ± 10
	HWI	27 ± 5	39 ± 9	26 ± 7	36 ± 8
	Trial × Time × CO_2_, *P *= 0.19: Trial × Time, *P *= 0.52: Trial × CO_2_, *P *= 0.62: Time × CO_2_, *P *= 0.41: Trial, *P *= 0.19: Time, *P *= 0.40: **CO_2_, *P *< 0.01**				
ICA_D_ (mm)	CON	5.03 ± 0.51	5.16 ± 0.47	5.08 ± 0.47	5.15 ± 0.51
	HWI	5.03 ± 0.47	5.17 ± 0.47	5.11 ± 0.58	5.16 ± 0.55
	Trial × Time × CO_2_, *P *= 0.59: Trial × Time, *P *= 0.84: Trial × CO_2_, *P *= 0.87: Time × CO_2_, *P *= 0.24: Trial, *P *= 0.74: Time, *P *= 0.44: **CO_2_, *P *< 0.01**				
ICA_CVC_ (ml min^−1^ mmHg^−1^)	CON	4.3 ± 1.3	5.8 ± 1.8	4.3 ± 1.3	5.5 ± 1.2
	HWI	3.9 ± 1.0	5.5 ± 1.5	4.1 ± 1.7	5.5 ± 2.3
	Trial × Time × CO_2_, *P *= 0.35: Trial × Time, *P *= 0.98: Trial × CO_2_, *P *= 0.85: Time × CO_2_, *P *= 0.67: Trial, *P *= 0.59: Time, *P *= 0.55: **CO_2_, *P *< 0.01**				
MCA_V_ (cm s^−1^)	CON	64 ± 17	82 ± 22	61 ± 18	76 ± 19
	HWI	56 ± 16	74 ± 21	49 ± 16	64 ± 20
	Trial × Time × CO_2_, *P *= 0.82: Trial × Time, *P *= 0.13: Trial × CO_2_, *P *= 0.90: Time × CO_2_, *P *= 0.06: **Trial, *P *= 0.04**: **Time, *P *= 0.02**: **CO_2_, *P *< 0.01**				
MCA_CVC_ (cm s^−1^ min^−1^ mmHg^−1^)	CON	0.8 ± 0.2	1.0 ± 0.2	0.7 ± 0.2	0.9 ± 0.2
	HWI	0.7 ± 0.2	0.8 ± 0.3	0.6 ± 0.2	0.8 ± 0.3
	Trial × Time × CO_2_, *P *= 0.48: Trial × Time, *P *= 0.89: Trial × CO_2_, *P *= 0.79: Time × CO_2_, *P *= 0.93: **Trial, *P *= 0.03**: Time, *P *= 0.21**: CO_2_, *P *< 0.01**				
Heart rate (bpm)	CON	60 ± 10	66 ± 12	58 ± 12	60 ± 11
	HWI	63 ± 11	68 ± 12	65 ± 11	65 ± 12
	Trial × Time × CO_2_, *P *= 0.51: Trial × Time, *P *= 0.08: Trial × CO_2_, *P *= 0.26: **Time × CO_2_, *P *= 0.03**: **Trial, *P *< 0.01**: Time, *P *= 0.09**: CO_2_, *P *= 0.03**				
MAP (mmHg)	CON	80 ± 12	84 ± 12	81 ± 8	85 ± 11
	HWI	84 ± 12	90 ± 14	81 ± 8	84 ± 8
	Trial × Time × CO_2_, *P *= 0.08: Trial × Time, *P *= 0.11: Trial × CO_2_, *P *= 0.32: Time × CO_2_, *P *= 0.29: Trial, *P *= 0.24: Time, *P *= 0.39: **CO_2_, *P *< 0.01**				
$P_{\mathrm{ET}O_{2}}$ (mmHg)	CON	102 ± 3	128 ± 7	103 ± 4	127 ± 8
	HWI	103 ± 5	130 ± 4	106 ± 5	128 ± 7
	**Trial × Time × CO_2_, *P < *0.05**: Trial × Time, *P *= 0.65: Trial × CO_2_, *P *= 0.68: **Time × CO_2_, *P *= 0.04**: Trial, *P *= 0.11: Time, *P *= 0.66: **CO_2_, *P *< 0.01**				
$P_{\mathrm{ETC}O_{2}}$ (mmHg)	CON	41 ± 3	53 ± 3	40 ± 3	53 ± 3
	HWI	41 ± 3	53 ± 3	39 ± 3	51 ± 3
	Trial × Time × CO_2_, *P *= 0.83: **Trial × Time, *P < *0.01**: Trial × CO_2_, *P *= 0.93: Time × CO_2_, *P *= 0.39: Trial, *P *= 0.17: **Time, *P *< 0.01**: **CO_2_, *P *< 0.01**				

*Note*: Data are presented as means ± standard deviation. *n* = 15 for all data except MCA_V_ (*n* = 14). Data were collected during $\mathrm{CV}R_{CO_{2}}$ assessments performed before and 45 min after 60 min in 21°C air control (CON) and 39°C HWI. Analysed by linear mixed effects model (LMM) with Greenhouse–Geisser correction to assess the effect of Trial (CON and HWI), Time (Pre and Post), Hypercapnia (Baseline and CO_2_) and interaction (Trial × Time × Hypercapnia). Abbreviations: ICA_D_, internal carotid artery diameter; ICA_CVC_, internal carotid artery cerebrovascular conductance; ICA_Q_, internal carotid artery blood flow; ICA_V_, internal carotid artery blood velocity; MCA_CVC_, middle cerebral artery cerebrovascular conductance; MCA_V_, middle cerebral artery blood velocity; MAP, mean arterial blood pressure; $P_{\mathrm{ETC}O_{2}}$, partial pressure of end‐tidal carbon dioxide; $P_{\mathrm{ET}O_{2}}$, partial pressure of end‐tidal oxygen.

**FIGURE 3:**
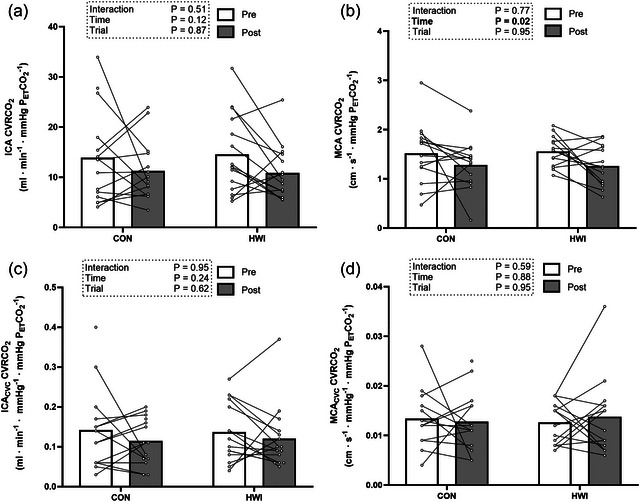
Internal carotid artery cerebrovascular reactivity to carbon dioxide (ICA $\mathrm{CV}R_{CO_{2}}$, a), middle cerebral artery cerebrovascular reactivity to carbon dioxide (MCA $\mathrm{CV}R_{CO_{2}}$, b), internal carotid artery cerebrovascular conductance reactivity to carbon dioxide (ICA_CVC_ $\mathrm{CV}R_{CO_{2}}$, c) and middle cerebral artery cerebrovascular conductance reactivity to carbon dioxide (MCA_CVC_ $\mathrm{CV}R_{CO_{2}}$, d) before and 45 min after 60 min in 21°C air (CON) and 39°C hot water immersion (HWI). Mean values before (Pre) and 45 min after (Post) are indicated by white (CON) and grey (HWI) bars, respectively. Open circles represent individual data. Analysed by linear mixed effects model (LMM) with Greenhouse–Geisser correction to assess the effect of Time (Pre and Post), Trial (CON and HWI) and interaction (Time × Trial).

Cerebrovascular reactivity was not different between HWI and CON (Figure 3). ICA $\mathrm{CV}R_{CO_{2}}$ did not change after either CON (pre: 13.9 ± 9.2 to post: 11.3 ± 6.1 mL min^−1^ mmHg^−1^) or HWI (pre: 14.6 ± 7.9 to post: 10.9 ± 5.4 mL min^−1^ mmHg^−1^; Interaction *P* = 0.65, Figure 3a). In addition, no interaction or main effect of trial was observed for MCA $\mathrm{CV}R_{CO_{2}}$ (Figure 3b), ICAcvc $\mathrm{CV}R_{CO_{2}}$ (Figure 3c) or MCAcvc $\mathrm{CV}R_{CO_{2}}$ (Figure 3d). Irrespective of the trial, MCA $\mathrm{CV}R_{CO_{2}}$ was lower after the interventions than before (Figure 3b, main effect of time, *P* = 0.02). This was not observed for ICA $\mathrm{CV}R_{CO_{2}}$, ICA_CVC_ or MCA_CVC_ $\mathrm{CV}R_{CO_{2}}$. The change in ICA $\mathrm{CV}R_{CO_{2}}$ pre‐ and post‐intervention was similar for participants who completed the CON trial first and HWI trial second (*n* = 7, CON; −6.2 ± 10.9 ∆ml min^−1^ mmHg^−1^ vs. HWI; −6.2 ± 7.8 ∆ml min^−1^ mmHg^−1^, *P* = 0.78) and those who completed the HWI trial first and CON second (*n* = 8, HWI; ‐0.8 ± 5.5 ∆ml min^−1^ mmHg^−1^ vs. CON; 2.9 ± 3.9 ∆ml min^−1^ mmHg^−1^, *P* = 0.16).

## 4 DISCUSSION

This study determined the effect of HWI on cerebral haemodynamics and subsequent $\mathrm{CV}R_{CO_{2}}$, which is an index of cerebrovascular function. To our knowledge, this is the first study to have utilised duplex ultrasound to volumetrically assess $\mathrm{CV}R_{CO_{2}}$ following a single session of whole‐body passive HWI. The main findings were that, in comparison with time‐of‐day matched control, HWI did not increase ICAQ or ICASR during the HWI, nor did 60 min of HWI influence subsequent CBF or $\mathrm{CV}R_{CO_{2}}$. In comparison with CON, HWI lowered arterial blood pressure and consequently ICACVC was increased to maintain blood flow to the brain.

### 4.1 Cerebral haemodynamic responses during whole‐body HWI

In contrast to the hypothesis, HWI did not increase ICA_Q_ or shear rate compared to CON; however, CBF was maintained during the whole‐body HWI despite large reductions in MAP. These findings contrast with previously observed elevations in ICA_Q_ during HWI (Gibbons et al., 2021; Worley et al., 2025) but support data that CBF can be maintained during heat stress (Watanabe et al., 2024). Given the sensitivity of the cerebrovasculature to changes in arterial carbon dioxide, lower $P_{\mathrm{ETC}O_{2}}$ during HWI compared to CON at 40 and 60 min time points in our study could have attenuated the CBF response to HWI. CBF is sensitive to arterial carbon dioxide, so avoiding hypocapnia is important to prevent reductions in CBF during heat stress. Hyperthermia‐induced hypocapnia typically occurs when *T*_c_ increases by more than 1°C from thermoneutral baseline (Bain et al., 2015), where the activation of temperature‐sensitive neurons within the medulla and carotid body initiates hyperventilation, increasing alveolar gas exchange (Gibbons et al., 2022). By design, our 39°C water immersion protocol was set to avoid excessive increases in *T*_c_ so that hyperthermia‐induced hypocapnia would be minimal. However, $P_{\mathrm{ETC}O_{2}}$ was lower during HWI than CON at 40 and 60 min, initiated before *T*_c_ exceeded a 1°C rise from the thermoneutral baseline. Impeding evaporative heat loss during exercise appears to increase the ventilatory sensitivity to carbon dioxide (Foster et al., 2023), so hyperthermia‐induced hypocapnia may be activated earlier during water immersion to the neck, which nearly completely impedes evaporative heat loss, compared to the sternal notch. This theory may help explain the decrease in $P_{\mathrm{ETC}O_{2}}$ in our study, whereas Gibbons et al. (2021), who utilised water immersion to the sternal notch, observed no decrease in $P_{\mathrm{ETC}O_{2}}$ at a 1°C rise in *T*_c_. The incorporation of an isocapnic condition for future research would help elucidate the contribution of $P_{\mathrm{ETC}O_{2}}$ to CBF responses to HWI.

Arterial blood pressure is a principal regulator of CBF, via cerebral perfusion pressure (Willie et al., 2014), so another possible explanation for the absence of an increase in ICA_Q_ in the current study compared to previous studies is a greater reduction in MAP during the immersion in our study (17 mmHg lower than CON), compared with Gibbons et al. (2021) (12 mmHg lower) and Worley et al. (2025) (2 mmHg lower). In addition, the hypotensive effect of HWI over time was driven by reductions in DBP, not SBP, as *T*_c_ increased. The larger hypotensive effect in our study may be due to experimental differences in water immersion depth compared to previous studies by Gibbons et al. (2021) and Worley et al. (2025). Both previous studies utilised water immersion to the sternal notch; in the present study, the participants were immersed in water up to the neck. The deeper water immersion would maximise skin surface area in contact with hot water, thus increasing the rate of heat exchange. This may result in more severe reductions in systemic vascular resistance, facilitating cutaneous blood flow to the skin and reducing arterial blood pressure in the heat (Crandall & Wilson, 2015). Consistent with this premise, a greater reduction in systemic vascular resistance is seen during HWI than sauna (Atencio et al., 2025), and greater water immersion depths result in greater reductions in DBP (Menzies et al., 2025).

### 4.2 The influence of whole‐body HWI on subsequent cerebrovascular function

In contrast to the hypothesis, the data show no influence of HWI on subsequent cerebrovascular function, as assessed by $\mathrm{CV}R_{CO_{2}}$. This supports previous work showing that HWI does not acutely influence post‐HWI cerebrovascular reactivity when assessed using TCD measures of the MCA (Worley et al., 2021, 2025) and provides additional data for this observation using volumetric‐based extracranial blood flow measures within the ICA. Similarly, gCBF, ICA_Q_ and VA_Q_ were unchanged following HWI compared to CON interventions.

A plausible explanation for these outcomes is that HWI was unable to significantly increase ICA_Q_ and ICA_SR_ during the HWI intervention. Interventions that acutely increase laminar blood flow and shear rate promote endothelial cell alignment (Wang et al., 2013), inhibit endothelial cell proliferation (Kadohama et al., 2007) and improve endothelial NO synthase phosphorylation (Casey et al., 2017), increasing vasoactive molecules and subsequently enhancing vascular function (Hodges et al., 2018). For example, exercise and intermittent hypoxia protocols that increase cerebral shear rate have been shown to subsequently enhance cerebrovascular function in a shear‐dependent manner (Iwamoto et al., 2020; Sakamoto et al., 2024). Furthermore, evidence in the peripheral vasculature indicates heat‐induced increases in blood flow and shear rate as a primary stimulus for both short (Cheng et al., 2019; Coombs et al., 2021; Thomas et al., 2017) and longer‐term (Brunt & Minson, 2021; Cullen et al., 2020) improvements in peripheral vascular function following heat therapy.

Another contributing factor to the lack of change in cerebrovascular function post‐HWI may relate to the specific assessment of cerebrovascular reactivity employed in our study. $\mathrm{CV}R_{CO_{2}}$ differs mechanistically from that of other cerebrovascular assessments, namely cerebral shear‐mediated dilatation (cSMD) (Carr et al., 2022; Hoiland et al., 2022). Like flow‐mediated dilatation (FMD) in peripheral arteries, cSMD is linked with endothelium‐dependent dilator pathways and the shear‐mediated production of NO; however, steady‐state $\mathrm{CV}R_{CO_{2}}$ is not endothelium‐dependent (Hoiland et al., 2022). Instead, $\mathrm{CV}R_{CO_{2}}$ is a non‐selective assessment of cerebrovascular vasoreactivity and therefore may not be sensitive to NO‐dependent changes in cerebrovascular function post‐HWI. As peripheral endothelium‐dependent function (FMD) can be enhanced following HWI even in the absence of a rise in shear rate during heat therapy (Coombs et al., 2021), it is possible that cerebral endothelium‐dependent function, that is, cSMD, may be improved following heat therapy even in the absence of a rise in cerebral shear rate during HWI.

A main effect of time was observed for MCA $\mathrm{CV}R_{CO_{2}}$, where MCA $\mathrm{CV}R_{CO_{2}}$ was lower post‐intervention compared to pre, irrespective of the trial. In contrast, this effect was not observed in the ICA. These differences may be explained by differences in vessel anatomy and physiology (Koep et al., 2022) and the methods used to measure blood velocity and blood flow in these vessels during the $\mathrm{CV}R_{CO_{2}}$ (Ainslie & Hoiland, 2014; Al‐Khazraji et al., 2021). Indeed, the MCA $\mathrm{CV}R_{CO_{2}}$ observation may be due to the interaction of MAP on the $\mathrm{CV}R_{CO_{2}}$ response, as the time effect for MCA $\mathrm{CV}R_{CO_{2}}$ was not evident when accounting for alterations in MAP with MCA_CVC_ $\mathrm{CV}R_{CO_{2}}$ calculations. Decreased vascular tone may have occurred across the trial (Panza et al., 1991), resulting in differential MCA diameter responses during hypercapnia, but these changes cannot be detected using TCD methods (Ainslie & Hoiland, 2014; Al‐Khazraji et al., 2021).

### 4.3 Applied implications and perspectives

During HWI, there was a substantial reduction in SBP, DBP and MAP that was evident upon immersion and became more pronounced during the HWI intervention. To compensate for the large reductions in MAP and maintain blood flow to the brain during HWI compared to CON, cerebral autoregulation mitigated the magnitude of arterial hypotension with a compensatory rise in ICA_CVC_. The large reductions in blood pressure during the immersion (17 mmHg) exceed values considered clinically meaningful (>5 mmHg) (Canoy et al., 2022) and suggest HWI may have some therapeutic value given that hypertension is the largest risk factor for stroke (Lawes et al., 2004) and a primary risk factor for cognitive impairment and dementia risk (Ishikawa et al., 2021; Kilander et al., 1998; Skoog et al., 1996). Moreover, our study provides evidence that large blood pressure reductions can be achieved with lower, more tolerable water temperatures (39°C) than have typically been used in previous studies (40–40.5°C) (Pizzey et al., 2021). Our thermal sensation, comfort and tolerance findings help highlight that HWI can be well tolerated, which is important if people are to engage regularly with HWI, and HWI is to be an effective method to help promote cardiovascular health benefits. Careful comparison of the methods and findings of the current study and previous research (Gibbons et al., 2021; Worley et al., 2025) suggests that subtle differences in water immersion depth and temperature influence CBF and blood pressure responses, where smaller increases in CBF and larger reductions in blood pressure might be expected with deeper HWI. Confirmation of this supposition by future research is needed to help develop evidence‐based guidance to maximise the health benefits of HWI.

### 4.4 Methodological considerations

Cerebrovascular reactivity was assessed 45 min after HWI and CON interventions, which is an important methodological consideration. In previous assessments of cerebrovascular function following intermittent hypoxia or exercise, the cerebrovascular assessments are typically initiated <25 min after the experimental intervention (Iwamoto et al., 2020; Sakamoto et al., 2024) or immediately and ∼60 min after the intervention (Burma et al., 2020; Sakamoto et al., 2021; Worley et al., 2025). Testing vascular function immediately following HWI is potentially problematic due to altered baseline haemodynamics. Cerebrovascular reactivity was chosen to be tested 45 min post‐HWI, as much of the post‐heat stress peripheral vascular function research has been conducted 30–60 min after heat therapy (Gravel et al., 2019; Romero et al., 2017; Thomas et al., 2016), and as previous research suggests, a biphasic pattern in vascular function following shear‐increasing interventions, where function is reduced immediately after, and then improved (Dawson et al., 2013)

Decreases in plasma volume and subsequent haemoconcentration lead to an increase in blood viscosity, which influences shear‐mediated processes (Carr et al., 2023). Although we did not measure blood viscosity directly, we did not observe differences between HWI and CON for Hct or plasma volume changes, and therefore, it is unlikely that blood viscosity was different between CON and HWI. Therefore, the absence of direct blood viscosity measures was unlikely to have influenced the outcome of $\mathrm{CV}R_{CO_{2}}$ before and after CON and HWI, respectively.

Our study included only young, healthy and recreationally active adults. Cerebrovascular function generally declines with age (Ainslie et al., 2008; Zimmerman et al., 2021) alongside the simultaneous rise in cerebrovascular disease risk (Ovbiagele et al., 2013). The identification and study of suitable non‐pharmacological interventions capable of improving cerebrovascular function and health is of vital importance. Heat therapy has the potential to improve cardiovascular function in physically inactive individuals and populations with cardiovascular co‐morbidity (Brunt et al., 2016; Ely et al., 2019; Roxburgh et al., 2025). Future research is warranted to examine the effects of HWI on cerebrovascular responses of older and diseased cohorts to better establish the feasibility and clinical value of HWI.

### 4.5 Conclusion

A single session of HWI reduced blood pressure and increased ICA_CVC_ to maintain blood flow to the brain. Nevertheless, HWI did not increase ICA_Q_ or ICA_SR_ or subsequent cerebrovascular function, as assessed by gCBF or $\mathrm{CV}R_{CO_{2}}$.

## AUTHOR CONTRIBUTIONS

Conceived and designed the work: Samuel F. Leaney, Justin S. Lawley, Geoff B. Coombs, Jonathan P. Moore and Samuel J. Oliver. Drafted the work, with all remaining authors reviewing and providing critical feedback important for intellectual content: Samuel F. Leaney and Samuel J. Oliver. All authors contributed to the acquisition, analysis or interpretation of data for the work. All authors have approved the final version of the manuscript and agree to be accountable for all aspects of the work in ensuring that questions related to the accuracy or integrity of any part of the work are appropriately investigated and resolved. All persons designated as authors qualify for authorship, and all those who qualify for authorship are listed.

## CONFLICT OF INTEREST

The authors declare no conflict of interest.

## OPEN RESEARCH BADGES

This article has earned an Open Data badge for making publicly available the digitally‐shareable data necessary to reproduce the reported results. The data is available at https://doi.org/10.6084/m9.figshare.29600135.v1.

## Data Availability

The data that support the findings of this study are openly available in Figshare, available at https://doi.org/10.6084/m9.figshare.29600135.v1
